# From erosion to fluency: reversing language shift in Chinese Australian households

**DOI:** 10.3389/fpsyg.2025.1553439

**Published:** 2025-02-28

**Authors:** Yining Wang, Jie Zhang

**Affiliations:** ^1^School of Foreign Studies, Guangxi Minzu University, Nanning, Guangxi, China; ^2^School of Foreign Languages, Zhongnan University of Economics and Law, Wuhan, Hubei, China; ^3^Faculty of Medicine, Health and Human Sciences, Macquarie University, Sydney, NSW, Australia

**Keywords:** Australia, immigration, heritage language, heritage bilingualism, 1.5 generation

## Abstract

Since the late 20th century, China-born population has emerged as the third largest source of permanent immigrants to Australia. This study aims to explore the dynamics of heritage bilingualism of twenty-five 1.5-generation Chinese-Australian adolescents and young adults, a cohort that is often overlooked in migration studies. Through family questionnaires, semi-structured interviews, field observations, and linguistic samples, the study explores how the age at migration influences language attitudes, proficiency performance, cultural identity, and socialization patterns among three age-of-migration cohorts. While the study confirms a common trend of language erosion across all age cohorts, it distinctively delineates the varying degrees of language attrition specifically associated with the age at migration. Meanwhile, the research spotlights exceptional cases of maintained heritage language fluency, underscoring how family strategies, child agency, educational policies, and literary engagement are crucial in combating language erosion and fostering heritage language proficiency. The finding underscores the importance of understanding the unique linguistic journeys across age-of-migration groups to better support their language development and maintenance. It provides valuable insights for families, educators, and policymakers working to sustain minority languages within a dominant English-speaking environment.

## 1 Introduction

Australia, a prominent destination for immigrants globally, had 29.5% of its population born overseas as of 2022 (Australian Bureau of Statistics, 2022). Among these immigrants, China-born individuals constitute the third-largest source, accounting for 2.3% of the total population (Australian Bureau of Statistics, 2022). In New South Wales, the most populous state, 38.6% of students in government schools in 2024 have a first language other than English, with Chinese background students making up 15.5% of this non-English speaking cohort (NSW Department of Education, 2024). Despite the Australian government’s linguistic diversity policies (Chen and Zhang, 2014), the dominance of English often leads to the weakening of minority languages (Hornberger, 2008). Mandarin is the most widely used language in Australian households after English but 62.7% of its speakers are first-generation Chinese immigrants (Australian Bureau of Statistics, 2021), indicating substantial challenges in maintaining and transmitting the heritage language among subsequent generations of Chinese Australians.

Language and migration inquiries often focus on immigrant children born in the host country or consider immigrant children as a homogeneous group (see Jeon, 2020; Shen and Jiang, 2021), overshadowing the unique experiences of the 1.5-generation—individuals who migrated during their childhood or adolescence (Venturin, 2019). This demographic, which finds itself neither fully integrated with their first-generation parents nor completely assimilated with their locally born peers (Choe et al., 2020), necessitates a more nuanced analysis of their engagement with both their heritage and societal languages, as well as their evolving identities and social affiliations. Existing literature on heritage bilingualism highlights minority children’s language shift and erosion as a pervasive trend (e.g., Nguyen, 2022; Tannenbaum and Yitzhaki, 2016), yet it seldom elucidates successful models of language preservation due to their rarity. While it acknowledges the influence of age of migration on language proficiency (Jee, 2018), it does not fully examine how precise ages at migration are linked to specific language proficiency potentials. This study aims to fill this gap by providing an in-depth analysis of heritage bilingualism patterns among 1.5-generation Chinese immigrants across a range of migration ages (4–13). It seeks to determine the association between various ages of migration and bilingualism patterns and to investigate the potential for fluency development that may counter the widely acknowledged attrition in heritage language proficiency. By examining both the overview picture and individual cases within each age cohort, this study illuminates the forces that resist intergenerational language discontinuity, shedding light on the preservation of minority heritage languages in a society where the mainstream language serves as the exclusive medium of instruction.

## 2 Theoretical framework of heritage language bilingualism

### 2.1 Heritage bilingualism and subtractive dynamics

Heritage (language) bilingualism refers to the linguistic phenomenon where individuals are raised with a non-dominant and minority language, known as the heritage language (HL), within immigrant homes or diasporic communities, while acquiring the dominant societal language in social and school domains (Rothman, 2009). These individuals, known as heritage speakers, typically acquire their HL as their first language during early childhood and encounter the societal majority language, either simultaneously in a bilingual context or sequentially as a second language (L2) (Montrul, 2023). HL bilinguals are not a uniform group, and they exhibit a significant diversity of bilingual competencies. This spectrum ranges from balanced bilingualism, where each language is used with equal proficiency and serves distinct communicative functions, to asymmetrical bilingualism, where one language becomes dominant, often influenced by factors such as societal dominance or limited exposure to the HL (Benmamoun et al., 2013). For speakers of minority languages, the acquisition of the majority language as a second language very often results in subtractive bilingualism, a scenario where individuals’ proficiency and use of their native or HL diminish as they adopt the societal majority language (Nguyen, 2022). This linguistic shift can progress to monolingualism, particularly among the third generation of immigrants, as societal language dominance often results in the marginalization and eventual loss of the HL (Portes and Hao, 2002). Fillmore (2000) observed that in the process of subtractive bilingualism, children from diverse backgrounds typically reach a stage in middle to late childhood where they become predominantly English-speaking or even monolingual in English.

### 2.2 Family and educational engagement in heritage language preservation

Many sociolinguists (Smith-Christmas, 2016; Spolsky, 2012; Tannenbaum, 2012) contend that language practice in the home is the key determinant in whether a language will persist through generations. Other scholars (Canagarajah, 2008; Curdt-Christiansen and La Morgia, 2018) argue that, in environments where a dominant language prevails, the family’s role is limited, lacking the autonomy to take independent action, especially when they are part of a linguistic minority within a multiracial and multilingual context. Even when families adhere to the “One Parent, One Language” approach—where each parent consistently speaks a different language to the child—children often develop into receptive rather than productive bilinguals, meaning they can understand the non-dominant language to a certain degree but not actively use it (Döpke, 1992, 1998, cited in King and Fogle, 2006). While the family serves as the primary domain in preserving HLs through affective, cognitive, and interactional support, this familial involvement alone does not provide adequate linguistic breadth, especially given the limited structured education access (Chen et al., 2024; Mu and Dooley, 2015). This limitation hinders young speakers from developing literacy, academic language, vocabulary expansion, and exposure to complex linguistic structures commonly found in different registers (Benmamoun et al., 2013). HL literacy, which uniquely (re) connects individuals and their families with life events of personal, symbolic, or social relevance in diverse ways, is often the least developed skill among HL speakers (Lee, 2024), and the first victim of language erosion (Tse, 2001). Given these challenges, enhancing family engagement in HL education should involve advocating for participative actions within curricula and classroom settings to effectively support HL development (Melo-Pfeifer, 2015). The remarkable progress of Chinese HL education in Malaysia can be largely attributed to the resilience and determination of the Chinese community in preserving their linguistic and cultural identity, as well as a level of integration of Chinese education within the national education framework, making Malaysia home to the most comprehensive Chinese-language education system in Southeast Asia (Kuang and Ling, 2015; Tan, 2021). However, the wide lack of success in HL education is prominently manifested in educational systems that do not recognize or support the HL, resulting in the loss of linguistic diversity and cultural heritage (Higby et al., 2023). Thus, a holistic approach to understanding language development in heritage bilingual children should take into account the complex bioecological systems ranging from the child’s immediate environment, such as family and peers, to the broader community and societal influences such as institutions and schools (Chondrogianni, 2023).

### 2.3 Migration age and critical period hypothesis revisited in proficiency outcome

The timing of migration, relevant to the onset and duration of language acquisition, is a pivotal factor in determining varieties of linguistic competencies, patterns of language use, and overall integration into the host society (Chondrogianni, 2023; Haim, 2024; Montrul, 2023). Research has consistently shown that early migration can often facilitate a stronger acquisition of the majority language and potentially prompt a shift away from the HL, while later migration can support the maintenance of the HL, albeit with different levels of proficiency and usage of the majority language (Montrul, 2023). This can be largely attributed to the critical period hypothesis, which suggests that there is an optimal period in childhood for language acquisition (Abutalebi and Clahsen, 2020). The critical period hypothesis is initially proposed for evaluating the optimal time for native language acquisition (Lenneberg, 1967) and then embraced by second language acquisition research and applied to foreign language learning (Siahaan, 2022; Singleton and Leśniewska, 2021). Recent scholarly efforts have begun to directly investigate the age effects on the HL proficiency of immigrants, an area previously understudied (Ahn et al., 2017; Montrul, 2008). Building upon the critical period hypothesis, Montrul (2008, 2013) expands its application to encompass language loss or incomplete acquisition in HL learning, proposing that reduced or interrupted input during childhood is also crucial, particularly affecting areas such as accent, grammar, and vocabulary. The debate on the critical period for language proficiency is marked by a wide divergence regarding its boundaries, ranging from early childhood to adolescence, with significant figures like Penfield (Penfield and Roberts, 1959) suggesting a critical age post-nine and Lenneberg (1967) associating it with puberty while more recent studies vary greatly in determining the age when the critical period ends, proposing ages as early as 3–4 years (Guasti, 2002) and as late as 17 years (Hartshorne et al., 2018). Despite the age debates, it was traditionally assumed that once acquired, native language knowledge is relatively stable and that environmental factors play a secondary role in language development (Crain, 1991). However, language attrition research has largely documented the progressive forgetting and loss of native speaker proficiency in both children and adults in a bilingual context after years of reduction in native language use or prolonged disuse (Montrul, 2023). That means, using the HL less after a certain critical age also has dramatic consequences for language development (Montrul, 2023). It is acknowledged that the younger the bilingual is when input is reduced or interrupted, the higher the loss of the native language will be (Ahn et al., 2017; Montrul, 2008). Under the influence of the majority language, young immigrants largely underwent structural changes including simplification of grammatical systems, confusion in tense usage, and loss of certain linguistic features (Montrul, 2008; Polinsky and Scontras, 2020). The proficiency outcomes of bilingual competencies, particularly in HLs, exhibit considerable variation among individuals and across communities (Alshihry, 2024). In the context of Korean Americans, Montrul (2008) and Ahn et al. (2017) pinpointed the age range of 9–12 as the most crucial for susceptibility to first language loss and the inability to learn a second language at native levels. However, within the Korean-Australian demographic, Jee (2018) identified the same age cohort as the ideal 1.5 generation, exhibiting the most favorable attitudes and robust motivation towards learning Korean, when compared with younger cohorts. This suggests that, beyond the age-led cognitive maturation constraints (Birdsong, 2018), the interaction between the age of migration and language proficiency involves complex social factors such as motivation, attitudes, resources, opportunities, and socioeconomic status which are grounded in both the immediate and broader contexts in which bilingual children are raised (Chondrogianni, 2023).

## 3 Materials and methods

### 3.1 Research participants

This study draws from a broader ethnographic study involving 31 first-generation Chinese families (32 children and 27 parents) residing in Sydney, the most populous Chinese community in Australia, from June 2017 to May 2020, focusing on Chinese children migrating before age 13 (Wang, 2020). The current study selected children who were aged 10 and above at the time of the interview and had been in Australia for at least 2 years by the time of the interview. The sample selection was guided by two primary considerations: (1) Adolescents and young adults are better equipped than younger children to articulate their thoughts, particularly on subjects related to nuances of cultural identity. (2) A stay of over 2 years in Australia may provide ample opportunity for families to participate in Australian social life, become familiar with the Australian education system, and establish their language practices. Based on these criteria, the study included 25 Chinese-Australian adolescents as the core subjects and their parents (a total of 18 individuals) as co-participants.

These families immigrated to Australia between 2000 and 2016. The adolescents and young adults, who are the focal point of this study, were aged between 4 and 13 at the time of their immigration to Australia and, at the time of interview, their ages ranged from 10 to 24, placing them within the Australian educational framework at the primary, secondary, or tertiary level. At the initial interview, these families had resided in Australia for a period ranging from 2 to 18 years, with an average of over 7 years. All 18 interviewed parents were well-educated, with 12 holding bachelor’s degrees, 4 holding master’s degrees, and 2 holding doctoral degrees.

Recognizing the relevance of age of migration in language proficiency and identity, the initial data analysis led to the decision to divide the adolescents and young adults into three age-of-migration cohorts: 4–7, 8–10, and 11–13, considering their schooling experiences in China. In China, preschool education typically starts around age 3, and formal primary education begins at age 6. The children who migrated between ages 4–7 were grouped because the only child who migrated at age 7 (Ge Si) had not completed Year One in China. Thus, all children in this group had limited or no formal schooling in China, forming the youngest age-at-migration cohort in this study. The children who migrated between ages 8–10 were grouped because they had completed 1–4 years of primary education in China. This cohort has a certain foundation of Chinese proficiency, reflecting a middle stage of Chinese language development. The children who migrated between ages 11–13 were grouped because they had completed primary education in China. This cohort has a relatively high level of Chinese proficiency before migration, reflecting a more advanced stage of language development. Thus, the age brackets for the three cohorts were chosen to reflect the developmental stages and educational experiences relevant to language acquisition and HL maintenance (Table 1).

**Table 1:** Summary of participant demographics by cohort.

Age at migration	Numbers	Avg. age at migration	Avg. age at interview	Avg. years in Australia	Language of interview
Group 1 (ages 4–7)	12	5.3	12.9	7.6	English (9); English & Mandarin (2); Mandarin (1)
Group 2 (ages 8–10)	8	8.9	14.9	6.0	Mandarin (7), English (1)
Group 3 (ages 11–13)	5	12.6	21.0	8.4	Mandarin (5)

### 3.2 Data collection

The data for this study is derived from family questionnaires, semi-structured interviews, field notes (informal conversations, participatory observations), and language artifacts. The family questionnaires, designed in English, were completed by parents or adult children before the interview, aiming to understand the family’s language and educational background to adjust the content of the interview (see Appendix 1). Semi-structured interviews, lasting 1–2 h with each parent and 30 min to 1 h with each child, cover the linguistic, educational, and life experiences of Chinese-Australian youth before and after immigration, focusing on their HL maintenance, cultural identity, school education, and social interactions in Australia (see Appendix 2). Informal conversations and participatory observations were conducted during regular social interactions between the interviewer and the interviewed families, typically when the interviewer visited participants’ homes or attended family gatherings in locations such as playgrounds or parks. The observations took place in natural settings and varied in length, from brief, casual conversations of approximately 30 min to pre-arranged activities that extended over half a day. The bits and pieces perceived as relevant were noted down immediately after these events, focusing on documenting the daily linguistic and cultural life of family members, particularly children’s language use with peers, siblings, parents, and other adults. Collected artifacts primarily included children’s reading materials and writing samples in Chinese, as well as academic reports from Chinese language teachers. Mandarin was primarily used as the default language for parent interviews, while children were encouraged to converse in their proficient or comfortable language. Code-switching between Mandarin and English was common during the interviews. All names of participants are anonymized.

Approval was obtained before data collection, ensuring the study met ethical standards for research involving human subjects. Informed consent was obtained from both parents and child participants, with clear communication about the potential for future publication and assurances of confidentiality (Table 2).

**Table 2:** Profiles of case-studied children.

Name/Gender	Age/Year at migration	Age at initial interview	Grade started in Australia	School grade at interview	Years of residence	Language of interview
Xia Tian/Male	5/2012	10	Kindergarten	Year 3	5	English
Li Long/Female	5/2007	15	Kindergarten	Year 9	10	English and Mandarin
Xinan/Female	9/2004	23	Year 3	Uni 5th Year	14	Mandarin
Su Shan/Female	10/2015	23	Year 4	Graduate	13	Mandarin
Bai Lan/Female	13/2007	24	Year 8	Uni 3rd Year	11	Mandarin
Dai Qin/Male	13/2013	18	Year 9	Uni 2nd Year	5	Mandarin

### 3.3 Data analysis

This qualitative analysis commenced by looking into the family questionnaires to gather demographic information about the focal children, including their age at migration, age at interview, language attitudes, preferences, and use within familial and co-ethnic contexts. Employing a grounded theory approach—a data-driven, bottom-up methodology for qualitative research (Dalderop, 2024), the study hierarchically coded interview transcripts and fieldnotes to identify nuances in language attitudes, proficiency, and social interaction tendencies (Table 3).

**Table 3:** Thematic coding scheme on heritage language bilingualism trajectories.

First-level theme	Second-level theme	Third-level theme
Language attitude	Negative attitudes	Being difficult, being boring, being irrelevant
	Positive attitudes	Being Chinese marker, being family tie, being good investment
Language ability	Language loss	Daily communication, character range, reading, writing
	Language erosion	In-depth conversation, character range, reading, writing
	Language progress	In-depth conversation, character range, reading, writing
Language use	Language use with parents	Chinese dominant, English dominant, bilingual
	Language use with siblings	Chinese dominant, English dominant, bilingual
	Language use with co-ethnic peers	Chinese dominant, English dominant, bilingual

The process started with open coding, where interview transcripts and fieldnotes were segmented, and codes were assigned to identify key concepts and phenomena. For instance, statements like “I find Chinese is way too hard” were coded as “negative attitudes” and “being difficult,” while “He even forgot how to write the character “大 (big)” was coded as “language erosion” and “character range.” In the axial coding phase, these codes were grouped into broader categories and subcategories to explore relationships and patterns. For example, codes related to “positive attitudes” and “negative attitudes” were grouped under “language attitudes,” and further subcategorized based on specific aspects like “difficulty,” “boredom,” or “relevance” and “Chineseness.” Selective coding identified the core category or central phenomenon. In this study, the core category was “heritage language bilingualism trajectories,” and all other categories and subcategories were related to this central theme. Some cross-thematic data were recoded for multiple themes.

After consolidating the findings under these thematic umbrellas, the study delineated typical patterns of heritage bilingualism proficiency among the focal children, with particular attention given to exceptional cases that may deviate from common trends.

## 4 Results: heritage language bilingualism across ages of migration

### 4.1 Correlation of migration age with language attitude and socialization

The initial examination of questionnaire data provides insights into how the 1.5-generation view Chinese and English, both positively and negatively, and what languages they prefer to use when interacting with different social agents or in various contexts, which seems to be relevant to the age at which these children migrate (Table 4).

**Table 4:** Language attitudes and language use by age at migration.

Number	Name/Gender	Language attitudes (multiple selection)		Language use (single selection) (C: Chinese (dominant); E: English (dominant); C & E: Chinese & English)			
		Negative (difficult/boring/irrelevant)	Positive (investment/identity/social tool)	With parents	With siblings	With co-ethnic peers	With interviewer
Group 1 (Immigrated at ages 4–7)							
1	Jie Ke/Male	✓		C & E	E	E	E
2	Xia Tian/Male		✓	C & E	E	E	C & E
3	Mo Jie/Female	✓		E	E	E	C
4	Bei Ni/Female	✓	✓	E	E	E	E
5	Ge Bai/Male	✓		C & E	E	E	E
6	Mai Se/Male	✓		C & E	E	E	E
7	Li Long/Female		✓	C	E	E	C & E
8	Xin Di/Female	✓		E	E	E	E
9	Ai Neng/Male	✓		C & E	E	E	E
10	Kai Li/Female	✓	✓	C	/	E	E
11	Xie Xi/Female	✓	✓	C & E	/	E	E
12	Ge Si/Female	✓		C & E	E	E	E
Group 2 (Immigrated at ages 8–10)							
13	Xu Li/Male	✓		C	/	E	C
14	Yang Mei/Female	✓	✓	C & E	/	E	C
15	Xing Dan/Male	✓	✓	C	/	E	C
16	Ma Li/Female	✓		C & E	/	E	E
17	Xi Nan/Female	✓		C	E	C	C
18	Kao Wen/Male		✓	C	/	E	C
19	Su Shan/Female		✓	C	/	C	C
20	Ji Mi/Male	✓	✓	C	/	E	C
Group 3(Immigrated at ages 11–13)							
21	Sha Wen/Female		✓	C	/	C	C
22	Zheng Ying/Female		✓	C	/	C	C
23	Bai Lan/Female		✓	C	C	C	C
24	Na Na/Female		✓	C	/	C & E	C
25	Dai Qin/Male		✓	C	C	C	C

For language attitudes, Group 1, consisting of individuals who migrated at the youngest age of 4–7, predominantly holds negative views towards their Chinese HL, with 10 out of 12 participants (despite the overlaps of views) considering it difficult, boring, or irrelevant. This suggests that early immigration may lead to rapid assimilation into the host culture’s language, potentially at the expense of devaluing the HL. In contrast, Group 3, which migrated at the oldest age of 11–13, exhibits a uniform positive attitude towards their HL, indicating that later migration fosters a greater appreciation for the language’s role in cultural identity and community engagement. Group 2, consisting of individuals who immigrated at ages 8–10, displays a mixed set of attitudes, suggesting a transitional phase where the balance between negative and positive views is more equal, with six negative views versus five positive views.

When examining language use across different social contexts, the age of migration continues to play a significant role. For Group 1, half of the participants (6 out of 12) use a mix of Chinese and English when speaking with their parents, while four predominantly use English, and only two use Chinese more frequently, indicating a stronger shift towards the host country’s dominant language. Besides mixed language use, the English dominance is particularly evident in their interactions with siblings (7 out of 12), co-ethnic peers (8 out of 12), and the interviewer (9 out of 12), aligning with their more negative attitudes towards their HL as indicated above. For Group 2, though young people show a dominant preference for Chinese when interacting with parents (6 out of 8) and the interviewer (7 out of 8), they shift to English solely or predominantly when speaking with co-ethnic peers (6 out of 8). This variety in language use suggests a transitional phase where the balance between the heritage and host languages is being negotiated within different contexts across different interlocutors. Conversely, Group 3 migrants, predominantly use Chinese across all social contexts, highlighting their positive attitudes and a robust attachment to and use of their HL, suggesting a stronger maintenance of linguistic heritage after an average of 8 years in the host country.

Thus, the timing of migration is crucial in influencing how young people value and engage with their HL, identify with language-specific social groups, and justify their cultural attributes. The earlier the migration, the more pronounced the assimilation into the Australian language and culture, while later migrants maintain a stronger connection to the Chinese language and culture, reflecting a deeper investment in their linguistic legacy. These findings highlight the dynamic correlation of migration age with language attitude and socialization and underscore the need for targeted language preservation and cultural integration strategies that consider the unique experiences of adolescents who migrate at different developmental stages.

### 4.2 Heritage language erosion across age-of-migration cohorts

Through interviews and observational data, the young people’s HL performance is characterized by a common thread: a pervasive experience of loss, decline, or inadequacy in Chinese language use. However, the specific patterns and the extent of this erosion are markedly influenced by the age at which individuals migrated to Australia.

#### 4.2.1 Fluency loss among 4–7-year-old arrivals

Young people who migrated at ages 4–7 typically embrace English as their principal or exclusive medium of social interaction and they often refer to English rather than Chinese as “my own language,” evidencing a pronounced loss and deficiency in their Chinese HL skills. Some within this cohort struggle to fulfill daily communication needs in Chinese. The use or partial use of English in the family domain is common, as parents (e.g., Jie Ke’s mother) reflected, “Even though we tell them to speak Chinese, they unconsciously speak English again.” When asked why they replied in English when their parents spoke to them in Chinese, they often cited limited vocabulary as a barrier to expression, as expressed in “So, like, I cannot think of the right words to use.” (Bei Ni) or “I just cannot speak a full sentence” (Xin Di), revealing a significant gap in their linguistic repertoire across generations. English dominance is particularly evident in interactions with siblings and other Chinese-background peers, with common responses being “We are used to it” (Jie Ke) or “We are better at English” (Ai Neng). This default to English indicates comfort and proficiency with the language that is more aligned with their daily experiences and educational environment. Despite limited proficiency in Chinese oracy, their initial low-level reading and writing abilities are more susceptible to significant regression or non-development. Quotes such as “I’m still speaking Chinese, but I cannot physically write a lot of words” (Xie Xi) and “I do not think I would read and write.” (Xin Di) exemplify the difficulties young immigrants encounter in initiating their reading and writing skills in Chinese.

For the youngest migrants, their linguistic shift can progress to monolingualism. Their rapid adoption of English often results in substantial regression of Chinese oracy and potential loss of its literacy, highlighting the greatest risk to their linguistic heritage due to early immigration.

#### 4.2.2 Literacy attrition among 8–10-year-old arrivals

Those migrating at ages 8–10 also predominantly use English but maintain a functional level of Chinese within the family, as exemplified by Tian Xing’s mother’s statement, “When he looks at me, he naturally speaks Chinese; once he steps outside the home, it’s all English.” This scenario is emblematic of the contextualized language pattern for most individuals in this cohort. Despite regular use of Chinese with parents on a daily basis, they also refer to English as “my main language,” which they use to justify their resistance to Chinese language learning or to legitimize their preference for English in most social settings. Their English preference underscores their strong inclination to assimilate into an English-centric environment while demonstrating a readiness to forgo their HL investment. The erosion of their HL is also notable, characterized by a significant decline in complex speech and a clear downward trend in reading and writing abilities. Yang Mei’s declining oral expressions, exemplified by her well-intentioned but mistaken Spring Festival greeting to her grandparents —substituting “A desperate dog tries to jump over the wall (狗急跳墙, ‘Goujitiaoqiang’)” for “Good luck in the Year of the Dog (狗年大吉, ‘Gouniandaji’)”— have become a lighthearted source of family humor. The incident, mentioned by her mother, underscores the potential for misunderstandings and miscommunications when language skills are not consistently practiced and reinforced. This cohort’s reading and writing abilities have particularly suffered, with parents (e.g., Ma Li’s and Ji Mi’s mothers) noting a stark contrast to their children’s previous Chinese literacy, such as reading sophisticated Chinese stories such as “窗前的小豆豆 (Beneath the Tree in the Courtyard), engaging with advanced literature like “红楼梦 (The Dream of Red Mansion),” and writing lengthy essays such as 500-to-800-words. Parents have observed that their children’s previously established literacy skills appear to “vanish” or “become dormant” within a few years in Australia. Tian Xing’s mother even expressed that Tian Xing’s Chinese writing skills had deteriorated to the point where he might not be able to write even simple characters like “自行车” (bicycle). In the interview, she even challenged Tian Xing to write it, indicating the rapid loss of his previously acquired writing skills.

The middle group also exhibits subtractive bilingualism, evidenced by their weakening skills in more complex use of HL. They maintain a functional level of Chinese at home but experience a notable simplification in their communicative repertoire. They lean heavily towards English outside in all or most aspects of their lives. They face the risk of diminishing literacy skills in Chinese, marking a phase where the HL is increasingly overshadowed by the new dominant language.

#### 4.2.3 Chinese academic insufficiency among 11–13-year-old arrivals

In contrast, those migrating at ages 11–13 often exhibit a pronounced habitus of speaking Chinese with peers from the same ethnic background and view themselves as active members of the Chinese community. Despite demonstrating a high level of fluency in Chinese during interviews and having completed a large part of their subsequent education in Australia, they generally perceive their proficiency in both Chinese and English as “not good enough” because both have not measured up to that of “native” or “local” speakers. When evaluating their bilingual abilities, they tend to allocate each language to distinct domains: Chinese is often seen as a more comfortable language for casual communication and emotional expression, as well as a more habitual choice for leisurely reading. Conversely, they express a clear sense of inadequacy when it comes to using Chinese in formal essay writing and academic contexts. Statements like “My grammar is very poor” (Na Na), “My Chinese writing is like a translated version from English” (Bai Lan), and “I could not understand complex Chinese literature like ‘围城’ (Fortress Besieged)” (Sha Wen) highlight the complexity of their linguistic challenges. The intricacies of literary texts and the construction of sentences in essays, which require a profound understanding of language structure and cultural nuances, can be particularly daunting for those who migrated during high school years. The inadequacy they feel stems from their perceived shortcomings in grammar, writing, and literary comprehension, which are largely attributed to limited exposure to advanced Chinese literature and its cultural subtleties following a disruption in their Chinese language education. It should be noted that the tendency of high-school migrants to speak Chinese and associate with Chinese speakers may be reinforced by their awareness of linguistic marginalization in a migration context. Bai Lan articulates this perceived ‘otherness’, highlighting the social and linguistic segregation that high-school migrants often encounter, as she emphasized “Chinese students stick with Chinese, and local students with locals,” which reflects the broader obstacles to linguistic and cultural assimilation experienced by older migrants.

For those migrating at later ages, their bilingualism exhibits complexities that are domain-specific, with a notable (partial) loss of linguistic capacity in academic and literary registers. They show greater resilience in sustaining Chinese, yet grapple with unique challenges with grammatical structures, literary comprehension, and academic exchanges, reflecting the linguistic and cultural gap due to a disruption of Chinese language education at a more advanced stage. Therefore, the age at which individuals migrate to a new linguistic context plays a pivotal role in determining the trajectory of their bilingual development and the extent to which their HL is retained or eroded over time.

### 4.3 Heritage language fluency beyond age-of-migration limits

Among the varied patterns of language erosion, notable exceptions are observed in each age cohort, demonstrating high levels of HL fluency in both oracy and literacy. In families with children migrating between ages 4–7, parents of individuals like Xia Tian and Li Long express satisfaction with their children’s Chinese language skills, a sentiment that is notably uncommon among families with younger immigrants. Within the 8–10 age group, Xi Nan and Su Shan stand out for their ongoing development in Chinese reading and writing, uniquely preferring Chinese as the primary language for communication with their Chinese peers. In the age 11–13 migration cohort, Bai Lan and Dai Qin exhibit a deeper understanding of Chinese classical literature and a stronger inclination towards the inheritance of Chinese cultural elements. The following analysis will delve into these exemplary cases, aiming to uncover the factors and pathways that have facilitated their transcendence of age-related migration challenges.

#### 4.3.1 “We are proud of his/her Chinese”—Xia Tian’s and Li Long’s parents

When many 4–7-year-old arrivals grapple with unfavorable attitudes towards learning Chinese, as well as challenges in daily Chinese communication and character recognition, Xia Tian and Li Long, distinguish themselves through their fluent Chinese during interviews, their active engagement with Chinese literature, and sustained dedication to Chinese language practices. Their proficiency in their HL can be primarily attributed to the strategic and consistent support from their families.

Xia Tian’s command of Chinese is particularly represented by his broad reading scope, which encompasses children’s literature, scientific texts, and educational materials. His father classifies the Chinese books chosen for Xia Tian into different genres, such as “encyclopedias for scientific knowledge,” “social–emotional intelligence books akin to ‘Chicken Soup for the Soul’,” and “academic study materials.” Xia Tian attests to the beneficial impact of these books on his life, illustrating how they have guided him in managing interpersonal relationships, particularly in moments when he feels “wronged at school,” by offering strategies derived from his readings. His parents take pride in their family language planning and express high satisfaction with Xia Tian’s Chinese language progress and his collaborative spirit. Xia Tian’s father regards “selective and extensive reading” as the cornerstone of maintaining Chinese language proficiency, which not only solidifies Xia Tian’s linguistic and problem-solving abilities but also significantly bridges the cultural gap between parents “with a Chinese mindset” and children “acculturated in Australia.” When talking about the positive aspects of the family language policy, Xia Tian’s parents often use the phrases “a reasonable boy” and “he is always willing to listen to parents’ advice” to express their sense of contentment. Through emotional, intellectual, and interactional support with heritage reading resources, Xia Tian’s family emerges as the primary domain that fosters his initial steps and broadens his linguistic breadth. The case of Xia Tian illustrates that proactive parental engagement and strategic guidance in the HL process can nurture reciprocal agency between parents and children, essential for fostering a cooperative learning environment.

Against the backdrop of widespread unfavorable attitudes towards learning Chinese among peers, Li Long is remarkable for her unwavering passion and well-entrenched habits for mastering Chinese, as well as her achieved fluency in oracy and literacy. At the first interview, it was noted that Li Long had been engaged in Chinese language learning in Australia for a decade. Her parents played a pivotal role in encouraging and facilitating a variety of Chinese language programs for her, which included family reading plans, community Chinese schools, Australian Chinese association activities, extracurricular Chinese tutoring, and short-term study trips back to China. Li Long’s mother highlighted the importance of these efforts, stating:

1. Those Sunday classes and tutoring classes, although taught by volunteers and possibly not systematic, helped my daughter keep up with Chinese. The community school played a key role with professional teachers and systematic teaching. Moreover, two study trips in China were like intensive training, showing huge effects in a short time.

Li Long’s case exemplifies the collective impact of family, community, and the school system on successful HL retention among adolescents who migrate at a young age. This integrated approach to resource utilization can nurture enthusiasm and a sense of identification with Chinese culture among Chinese-Australian youth. Li Long’s mother notes that her daughter’s study experiences in China “particularly fostered a deep emotional connection to China.” Li Long herself underscores the relevance, stating, “China and Chinese are relevant to me.” Contrary to most of her peers who forsake Chinese and distance themselves from Chinese social circles, Li Long actively seeks out and engages with newly arrived Chinese children, forging social connections in Chinese.

In fact, among the common risks of severe attrition or language loss among those 4-7-year-old arrivals, Xia Tian and Li Long’s achievement, especially in the realm of literacy, is remarkable. Their cases provide empirical evidence supporting the family unit’s role as the primary domain for language transmission. Furthermore, their experiences demonstrate the efficacy of a varied selection of HL reading materials as a potent vehicle for exposing children to complex linguistic structures, which is vital for literacy development, especially in settings where structured educational access is constrained. Their narratives also underscore the vital importance of a synergistic approach that combines family, community, and school efforts in fostering a more comprehensive acquisition of the HL, which is crucial for enriching the cultural identity and boosting the academic potential within the context of childhood migration.

#### 4.3.2 “Few Chinese peers can indeed reach my level”—Xi Nan and Su Shan

Contrary to the prevalent trend of English language dominance and the subsequent decline in Chinese literacy among the 8–10-year-old immigrant cohort, Xi Nan and Su Shan demonstrated sustained progress in their Chinese reading and writing skills and engagement with the Chinese community, which highlights the pivotal role of child agency and reading habits language retention and advancement.

Su Shan’s achievement in Chinese literacy after immigration can be largely due to her engagement with the Chinese community and structured language programs in school. She opted for Chinese as a HL in high school, which provided a curriculum tailored to her background and prior knowledge of Chinese. As she recalled, “Because students who came before the age of 10 have the chance to choose the heritage course.” This structured program is instrumental in allowing her to engage deeply with the language, especially refining her writing skills.

Xi Nan’s deep affection for the Chinese language is particularly striking, as she articulated its beauty with a personal touch, “I really like it because I find the Chinese language very beautiful, especially classical Chinese.” Her fondness is not just theoretical; it permeates her routines and scholarly endeavors. This is evident in her frequent borrowing of Chinese literature from the community library, where she noted, “I keep borrowing books and still read traditional Chinese characters because there are many romance novels that are only available in traditional characters, not in simplified ones.” Her choice to engage with literature in Chinese, even in an English-speaking context, highlights a profound emotional and aesthetic bond with the language, as well as a deep-seated appreciation for the expressive richness of her HL. Xi Nan’s engagement with the Chinese language is further exemplified by her practices of “writing Chinese diaries” and “practicing Chinese calligraphy,” activities she deems essential for “not forgetting Chinese while living in Australia.” These efforts are further solidified by her academic endeavors in university, where she has chosen to study Chinese literature and poetry, even serving as the class representative for Chinese poetry.

Xi Nan and Su Shan have both navigated their social circles with a common thread preserving their HL and cultural ties. Despite being raised in Australia, their interaction with Chinese-speaking peers is continuous, reflected in their common practice of using Chinese in peer engagement, “There were two others who played with us in elementary school and middle school. We’ve been together ever since. We always speak Chinese with each other.” This illustrates the close-knit nature of their social circle, where language serves as a bridge to their cultural heritage. Su Shan emphasized the crucial role of her social circle in language maintenance, “I can keep my Chinese as good as it is because I have so many friends who speak Chinese.” Their proficiency in Chinese and familiarity with Chinese culture also enable them to engage with the broader Chinese community, as Xi Nan explains:

1. Because I also watch some Chinese TV dramas [...] whatever is popular, I watch them all, like 三生三世 (Eternal Love),’ ‘十里桃花 (Ten Miles of Peach Blossoms), ‘我的前半生 (My First Half of Life),’ I watch everything. That’s why I have a lot to talk about with them (new international students).

This quote reflects how Xi Nan’s social interactions often center around shared cultural interests and activities with her Chinese friends, such as discussing popular TV dramas. Indeed, the Chinese social circle not only provides a supportive environment where they can practice and refine their Chinese language skills but also offers them a sense of belonging and cultural continuity. Xi Nan and Su Shan place a high premium on their Chinese-English bilingual identity, viewing it as an asset that enriches their social and academic experiences. Xi Nan articulated this perspective, saying, “For me, both languages are very important, they allow me to communicate with a wider range of people and shape my way of social interaction and my attitude towards learning.”

Xi Nan and Su Shan’s narratives emphasize the complex interplay of factors like child agency, reading habits, social engagement, and structured language education in maintaining Chinese literacy and celebrating cultural roots among young immigrants. Despite living in an English-dominant environment for over 10 years, their commitment to Chinese language practices is commendable. They actively engage with Chinese literature, maintain friendships with Chinese speakers, and opt for Chinese language courses, which not only strengthen their bilingual proficiency but also enhance their social and academic experiences.

#### 4.3.3 “We want to be Chinese teachers in Australia”—Bai Lan and Dai Qin

Young people in the 11–13-year-old immigrant cohort all exhibit fluent oral proficiency and maintain extensive social networks within the Chinese community. Bai Lan and Dai Qin, in particular, have a strong foundation in Chinese literature and have incorporated Chinese education into their career aspirations. Like Xi Nan and Su Shan, Bai Lan and Dai Qin’s journey with Chinese language learning is marked by a high level of self-direction, as they both expressed, “Chinese is very important, and I have a clear plan for the future; I want to be a Chinese teacher in Australia..” This clear career planning also indicates that their mastery and love for the Chinese language should reach a deeper level of literary competence, which transcends the functional use of Chinese for daily communication. To achieve this, they opted for Chinese literature courses during their high school years, pursuing an in-depth understanding and appreciation of Chinese literature. Dai Qin reflected:

1. The reason my Chinese has improved is that I learned a lot of literary content in high school, such as the poetry of the Tang Dynasty, the lyrics of the Song Dynasty, and the operas of the Yuan Dynasty, and wrote many Chinese essays, including literary reviews and appreciations of modern novels like ‘围城 (Fortress Besieged),’ ‘骆驼祥子 (Rickshaw Boy),’ and ‘红高粱 (Red Sorghum).’ These foundations have been very helpful for my current university studies in Chinese.

Dai Qin’s linguistic journey underscores the indispensable function of school curricula in shaping the linguistic sophistication of adolescent immigrants. The structured educational environment plays a pivotal role in developing complex linguistic structures and expanding linguistic repertoires, particularly in the academic and literary registers of Chinese. Beyond individual initiative, the specialized courses and educational materials, hardly accessible outside of a formal educational setting, largely mitigated cultural and linguistic gaps that often arise from the disruption of Chinese education in children’s formative years, enabling an in-depth exploration of the richness of Chinese language and literature. Bai Lan and Dai Qin’s enthusiasm for Chinese and their ambition to become Chinese teachers not only reflect their profound identification with Chinese elements but also show their strong desire to pass on this cultural heritage. While English often dominates their academic and professional lives, Chinese remains a language of ethical insights and moral guidance, as Bai Lan said:

1. I find that Chinese culture is really vast and it’s taught me a lot about bigger ideas in life. In high school, taking Chinese as a heritage course was very enlightening. It covered life attitudes, values, and a sense of belonging, which helped me grow and understand things better.

The quote encapsulates the deep connection between language fluency, cultural identity, and personal growth, emphasizing language as a vital vehicle for conveying ancestral values and facilitating cultural continuity, despite geographical or generational distances. The experience of taking a Chinese heritage course in high school appears to have been a transformative journey, revealing the depth and breadth of Chinese philosophical legacy, which serves as a source of personal strength and enlightens broader life lessons. It suggests that language fluency is more than just grammatical correctness or vocabulary expansion; it’s about the ability to engage with cultural nuances and the wisdom encoded within a language.

Bai Lan and Dai Qin, through their steadfast preservation of their HL, are carving out a niche for the robust growth of their Chinese language skills within the English-centric mainstream culture, bridging the divide between their ancestral past and their present, and reaffirming their deep connection to their cultural roots. Their journey of being fluent and highly literate Chinese users, though driven by a high degree of self-direction, is a testament to the significance of structured learning environments like formal education on their language acquisition, career aspirations, and the formation of their cultural identity.

## 5 Discussion: from erosion to fluency

### 5.1 Age of migration in the variation of language erosion

The current study underscores the significant role of migration age on the different levels of Chinese language attrition and the varied patterns of heritage bilingualism. The correlation between migration age and reported proficiency outcome aligns with previous research suggesting that early migration facilitates a quick acquisition of the majority language, often to the detriment of the HL, while later migration tends to maintain higher levels of HL proficiency and heritage investment (Montrul, 2023). This study uniquely maps specific migration ages to the varying degrees of Chinese language decline in oral, reading, and writing skills, a dimension that is underrepresented in qualitative depictions of real-world language use. The distinct patterns of language erosion across different age cohorts of Chinese-Australian migrants are represented by the youngest migrants (ages 4–7) progressing towards monolingualism in English, to the middle cohort (ages 8–10) experiencing regression in complex oral expression and literacy, and the oldest migrants (ages 11–13) maintaining robust oral communication skills in Chinese but facing gaps in academic and professional Chinese. These varied trends reflect the differing impacts of disrupted mother tongue education at various developmental stages in early childhood, middle childhood, and adolescence.

This study supports the existence of a critical period for language acquisition, suggesting an optimal window in childhood for language acquisition (Abutalebi and Clahsen, 2020). Montrul (2008, 2013) and Ahn et al. (2017) extend the critical period hypothesis, conventionally reserved for first and second language acquisition (Lenneberg, 1967; Singleton and Leśniewska, 2021), to encompass the realm of language attrition and incomplete acquisition within HL education. They proposed that when in childhood input becomes reduced or interrupted is crucial, especially impacting areas like accent, grammar, and vocabulary (Montrul, 2008, 2016). The ages of 9–12 were identified as the pivotal period, being either the most vulnerable to first language loss or attrition (Ahn et al., 2017; Montrul, 2008) or the most conducive to maintaining and preserving the first language (Jee, 2018). Our study reveals a more nuanced view of language maintenance and shift by identifying a broader spectrum of critical migration periods between ages 4–13, each with unique vulnerabilities in Chinese HL proficiency.

Unlike much of the existing research that situates the examination of age factors in language acquisition within developmental psychology and focuses on cognitive flexibility or brain plasticity (Birdsong, 2018), this study delves into the socio-emotional factors and diverse linguistic challenges associated with the age of migration - the time when HL use declines and Chinese language schooling is disrupted. It reveals that children arriving at ages 4–7 quickly assimilate linguistically, a process eased by simpler communication demands in their early academics and greater perceived integration opportunities, potentially resulting in limited motives to care for their Chinese language skills. In contrast, those arriving at ages 11–13 face significant English language challenges from the outset, a result of the need to express complex ideas, master academic English, and handle a more rigorous curriculum in Australia. These language challenges are further exacerbated by feelings of linguistic otherness and racial marginalization, which might paradoxically reinforce their adherence to Chinese. The timing of immigration can markedly influence the societal context and linguistic environment for heritage bilinguals, which in turn affects both the quantity, such as the amount of exposure to their languages, and the quality, including the richness and complexity of language input that children with different immigration experiences receive (Chondrogianni, 2023). The study brings to light the impact of socio-emotional factors at various age stages on language use across different domains, an area not commonly addressed in linguistic studies but influences the extent and richness of language use (Pułaczewska, 2021). The varieties of HL bilingualism patterns highlight the dynamics and intricacy of language use and attrition among heritage speakers and underscore the importance of understanding their unique linguistic journeys across age-of-migration groups to better support their language development and maintenance.

### 5.2 Key factors reversing the erosion pattern

Despite the pervasive pattern of language erosion, the study identifies notable exceptions of fluent and highly literate heritage speakers across different age cohorts. These individuals, relative to their age peers, demonstrate a notable level of fluency (e.g., capacity for Chinese interviews) and literacy (e.g., comprehension of literature and/or writing essays) in Chinese HL, challenging the conventional narrative of language shift and loss. The developmental trajectories of these highly proficient HL users underscore the significance of four distinct facilitative factors: family language strategies as the foundation for language transmission, child agency as the driving force for linguistic interaction, institutional support as the guarantee for language sustainability, and literary engagement as the determinant for the ultimate attainment.

Within educational systems where the majority language, notably the global lingua franca English, serves as the sole medium of instruction, the family language environment emerges as a core domain (Spolsky, 2012), largely determining whether children can attain proficiency in their minority HL or risk losing their HL (Curdt-Christiansen and Hancock, 2014). Besides parental mere desires for HL transmission, what matters most regarding the efficacy of family language policy is “what parents do or do not do” to support their children’s HL proficiency (Li, 2006a, p. 29). This research, while emphasizing the importance of the family’s role, places a spotlight on identifying what specific measures families should take and how to ensure the continuity of the HL within the household. Exemplary early intervention models, such as Xia Tian’s family’s ‘selective extensive reading’ strategy, highlight the development of literacy, intelligence, personality, and intergenerational understanding. The synergetic use of familial, community, and school resources, as adopted by Li Long’s family, further fosters heritage language robustness within households. These early intervention cases stand out against the giving-up behavior observed in many families who often prioritize their children’s immediate success in English and related subjects, as also seen in various contexts such as Singapore (Curdt-Christiansen and Hancock, 2014) and the USA (Zhang, 2012). The persistence and commitment of these early intervention cases underscore the importance of diversity, consistency, and continuity in language sustainability. In a mainstream English culture such as Australia that largely lacks structured education for minority languages (Hornberger, 2008), the family’s engagement serves as the initial cradle and its persistence as the last bastion of language transmission for their young children.

Children’s agency, or their active role in family language decisions (Curdt-Christiansen, 2020) significantly sustains the continuity of family language policy. Xia Tian’s proactive participation in reading and his interaction with texts reflect the bidirectional efforts of parents and children in cultivating HL and bilingual abilities. Li Long’s transition from initial passivity (such as early resistance to attending community school) to actively engaging in HL classes (such as a voluntary request to study in China) ensures her sustained Chinese literacy practice over a decade. Later migrant children (e.g., Xi Nan, Su Shan, Bai Lan, and Dai Qin) independently pursue Chinese learning by connecting with Chinese speakers, studying literature, and practicing writing and calligraphy, highlighting how their initiative and motivation can sustain language acquisition and cultural transmission despite minimal parental involvement. This also illustrates the correlation between Chinese language proficiency and emotional ties to the motherland.

Academic input in the HL during the school-age period is a crucial dimension for input quality, as it introduces written language structures that are typically more complex and nuanced than those encountered in everyday spoken communication (Montrul, 2023), but a lack or deficiency in institutional support for minority languages is well-documented in various migrant contexts (Wang, 2024; Tannenbaum and Yitzhaki, 2016). This is particularly evident in English-centric societies such as Australia, where non-English language education is fragmented and proficiency levels are historically low (Hornberger, 2008). In the study, amidst the widespread trend of English overshadowing Chinese among both parents and children, Bai Lan and Dai Qin exemplify the significance of institutional resources by integrating Chinese language learning into their career plans and actively seeking out the limited opportunities available in high schools and universities to engage with Chinese literature courses. These specialized resources not only strengthen their command of Chinese within academic and literary contexts but also broaden their perspective on life with cultural wisdom, largely mitigating the lack of structured support in families and communities. The ongoing commitment to Chinese language maintenance among the case-studied children is significantly bolstered by systematic educational support, evidenced in their selected literary courses tailored for HL learners in Australia.

Literacy in the HL enables individuals to delve into intricate dimensions of linguistic artistry, grammatical structures, and sociocultural contexts, with implications for overall language growth that enhance but extend beyond everyday vocabulary for mere communication (Montrul, 2023). The exceptional accomplishments of these six immigrant children across different age groups collectively underscore the pivotal impact of their literacy development, which has been substantially supported by engaging literary works and tailored reading materials. Acquiring advanced HL literacy empowers them to bridge generational understanding and enhance problem-solving skills (e.g., Xia Tian), engage with the poetics of language and appreciate its aesthetic dimensions (e.g., Xi Nan), construct sophisticated arguments in formal settings (e.g., Dai Qin), enlighten life philosophies and construct their multiple identities (e.g., Li Long and Bai Lan), and fully participate in heritage community (e.g., Li Long, Xi Nan, Su Shan, Bai Lan, and Dai Qin). Particularly illustrative are the cases of Xia Tian and Li Long, showing how early intervention and steadfast reading practices foster young immigrants’ Chinese literacy, a skill often deemed unachievable by young immigrants. Xia Tian’s father’s strategic use of diverse literature, spanning science, social–emotional intelligence, lifestyle, and academics, has significantly enhanced Xia Tian’s linguistic breadth and cognitive growth, achieving a rare “harmonious bilingualism” (De Houwer, 2020) in immigrant families. Xi Nan and Su Shan’s appreciation of both classic and pop culture narratives underscores the crucial role of literary engagement in valuing cultural heritage and expanding social networks. For the later immigrants, Bai Lan and Dai Qin’s in-depth exploration of Chinese literature and philosophy in heritage classes not only refined their language skills but also shaped their future educational and vocational aspirations. Incorporating literary works into the curriculum can provide an optimal textual resource for HL learning in education systems where minority languages are not the main subject. Text exposure is vital for expanding vocabulary repertoires, understanding language registers and pragmatics across various contexts, and mastering written-language-specific structures (Montrul, 2023). Exposure to Chinese literary texts helps children overcome the limitations of mainstream HL education, leading to a more comprehensive understanding of Chinese and expanded career opportunities.

Previous studies, while advocating for family and institutional support in HL transmission (Romanowski, 2022; Zhang, 2012), have been vague in describing what constitutes a more ‘sufficient input’ and have inadequately addressed the types and extent of literacy practices that contribute to the development of robust HL proficiency. This study bridges the gap in current sociolinguistics by delineating the lived challenges of children who arrive in Australia at various ages and providing a detailed and nuanced understanding of the concrete measures that individuals, families, communities, and institutions can take to foster more complete heritage bilingualism. It offers clear evidence showing how families employ reading resources to broaden their children’s HL and knowledge, how children leverage literature books to enrich their literacy and popular programs to expand social circles, and how young adults benefit from their literary and academic language courses in schooling. The study underscores that the neglect of literacy support is a key factor leading to prevalent language attrition patterns and suggests that a lack of formal education in the HL limits heritage speakers’ linguistic repertoire and their ability to use the HL in diverse registers, particularly in formal and scholarly contexts. It advocates for a comprehensive approach that emphasizes consistency and persistence from all stakeholders to nurture the robust and sustainable growth of HLs. Indeed, the tendency towards attrition or subtractive bilingualism can be mitigated by practices and policies that support quality maintenance, considering family strategies and language courses designed to nurture heritage speakers’ literacy across different linguistic registers (Lubińska, 2024).

### 5.3 Influence of socio-economic status on heritage language maintenance

Socioeconomic status (SES) significantly impacts HL maintenance among diasporic communities, influencing both opportunities and challenges. The role of parental SES in their children’s HL achievement can vary across different academic studies and immigrant groups. For example, Chinese families with higher SES in the USA often have more resources and opportunities (e.g., access to community programs and educational materials) to support language practices, while those with lower SES face more challenges (e.g., limited time, financial constraints) (Liang and Shin, 2021). In contrast, Vietnamese families in Montreal show differences in HL maintenance based on migration reasons, with economic immigrants experiencing a more significant decline in their children’s Vietnamese language skills compared to political immigrants (Nina Le and Trofimovich, 2023). In this study, the families are likely to have a relatively high SES, given the parents’ educational background. This aligns with broader trends in immigrant communities, where higher parental education is often associated with greater knowledge and active involvement in raising bilingual children (De Cat, 2021; Li, 2006b). The high educational background and likely high SES of the parents in this study may have contributed to effective family language strategies, such as selective extensive reading and educational programs like study trips back to China (Section 4.3.1). However, not all parents were able to balance Chinese maintenance with English advancement, a challenge compounded by the unbalanced power relations of languages in mainstream society. Despite this, the study suggests that while not all socio-economically well-off parents prioritize HL education, the acquisition of high-level literacy skills in heritage bilingualism is closely related to parental educational background and SES.

### 5.4 Limitation and future direction

While this study offers valuable insights into the dynamics of heritage bilingualism and cultural identification across ages of migration among Chinese-Australian adolescents and young adults, it acknowledges certain limitations. The sample size, although representative, is relatively small, which may constrain the generalizability of the findings to the broader Chinese diaspora or other populations. Future research could benefit from expanding the sample and incorporating longitudinal studies that track language and identity development over a more extended period. Furthermore, combining proficiency assessments with standard measures to quantify language skills and juxtaposing these with the qualitative sociological findings could yield a more comprehensive view of language retention and attrition. By addressing these gaps, subsequent studies can contribute to a better understanding of the factors influencing HL bilingualism among immigrant populations in Australia and globally.

## 6 Conclusion

This study provides a comprehensive analysis of HL performance, heritage bilingualism, and cultural identification among Chinese-Australian adolescents and young adults, particularly focusing on the 1.5 generation. The research underscores the pivotal role of age at migration in predicting language attitudes, language habitus, bilingualism patterns, and socialization affinity. It reveals a pattern of language erosion across all age cohorts, with earlier migrants showing a stronger assimilation into Australian language and culture, and later migrants maintaining a more robust connection to the Chinese language and cultural identity. However, the study also identifies exceptional cases of HL fluency that defy the conventional trend of language erosion, highlighting the importance of family-based strategies, child agencies, language-in-education policies, and reading engagement. These findings suggest that with targeted support and engagement, it is possible to reverse the trend of language shift and develop a strong command of their HL and a deep appreciation for it. The study concludes that while the journey toward HL fluency is complex and multifaceted, it is not insurmountable, offering valuable insights for families, educators, and policymakers aiming to preserve and promote minority HLs in a dominant English-speaking context.

## Data Availability

The original contributions presented in the study are included in the article/Supplementary material, further inquiries can be directed to the corresponding author/s.
