# Supplementary material for: From erosion to fluency: reversing language shift in Chinese Australian households
Source: Front Psychol. 2025 Feb 28;16:1553439. doi: 10.3389/fpsyg.2025.1553439 (PMC11916325; doi:10.3389/fpsyg.2025.1553439)
Supplement: Supplementary file 1 [file Data_Sheet_1.docx]

**Appendix I Family questionnaire (a revised version)**

There are 3 main sections in this questionnaire. Section 1 is about some background information of parents. Section 2 mainly includes your family’s language and migration experiences. Section 3 is the general information of the focal child.

**Section 1 Background information**

*About yourself*

1. What’s your name? ______________________
2. What’s your relationship with the child? □ father □ mother
3. What’s your age? □ 20-30 □ 30-40 □ 40-50 □ 50+
4. What is/are your first language/s?

□ Mandarin □ Cantonese □ English □ Others (please specify):______________

1. In which year did you arrive in Australia? ________________
2. What is your highest academic qualification?

□Primary □ high/secondary □ Bachelor □ Postgraduate

1. What was your occupation prior to your migration?
2. What is your occupation now?

*About your spouse/partner*

1. What’s your name? ______________________
2. What is your age? □ 20-30 □ 30-40 □ 40-50 □ 50+
3. What is/are your first language/s?

□ Mandarin □ Cantonese □ English □ Others (please specify):_______________

1. In which year did you arrive in Australia? ________________
2. What is your highest academic qualification?

□Primary □ high/secondary □ Bachelor □ Postgraduate

1. What was your occupation prior to your migration?
2. What is your occupation now?

**Section 2 Language and migration experiences**

*About your family*

1. What is your current citizenship status / your visa type? _______________________
2. How many children do you have? ______________________________
3. How many people in total live in your household? ___________________
4. What are the other adults living together except parents?

□Grandparents □ Others (please specify):__________________

1. What language/s do parents speak to each other at home? ______________________
2. What language/s do parents speak to child? _______________________
3. What language/s does your child speak to parents? ______________________
4. What languages do other adults at home speak to child? ____________________
5. What language/s does your child speak to other adults at home? ______________________
6. What language/s does your child speak to his/her siblings? _____________________
7. Which suburb are you living in now? ________________________
8. What were the reasons for migration? (you may choose more than 1 answer)

□a. Work/business

□b. Your (or your spouse/partner’s) education

□c. Your children’s education

□d. Family reunion

□e. Better living condition

□f. Political reasons (please explain): ____________________________________

____________________________________________________________________

1. What is your contact mode?

Phone number: __________________________________

Email: _________________________________

**Section 3 General information of child**

1. What’s your child’s name?　＿＿＿＿＿＿＿＿＿＿＿＿
2. In which year was your child born? ______________________
3. In which year did your child arrive in Australia? _____________
4. In which type of school was your child prior to migration?

□ Chinese language/ public schools

□ Bilingual/ Chinese-English schools

□ Others (please specify):_______________

33. At what year/grade was your child prior to migration? ___________________

34. From which year/grade did your child start after he/she arrived in Australia? ___________

35. What books (languages) does your child read?

- Chinese books only
- English books only
- More Chinese books
- More English books
- Half-half

Others (please specify):________________

36. What movies/TV programs (languages) does your child watch?

- Chinese programs only
- English programs only
- More Chinese programs
- More English programs
- Half-half
- Others (please specify):______________

37. Which languages does your child use when talking with friends?

- Chinese only
- English only
- More in Chinese
- More in English
- Half-half
- Other languages (please specify):____________

**Additional questions (if applicable)**

38. Can you use some words to describe your feelings about learning Chinese (e.g., interesting, boring, difficult, unjoyful, and etc.)?

_______________________________________________________________________

39. Can you use some words to explain why it is important or unimportant to learn Chinese (e.g., family communication, future job, more friendship, irrelevant, and etc.)?

_______________________________________________________________________
