# Supplementary material for: From erosion to fluency: reversing language shift in Chinese Australian households
Source: Front Psychol. 2025 Feb 28;16:1553439. doi: 10.3389/fpsyg.2025.1553439 (PMC11916325; doi:10.3389/fpsyg.2025.1553439)
Supplement: Supplementary file 2 [file Data_Sheet_2.docx]

**Appendix II Interview framework**

**Interview Guides (for parents)**

**Phase 1** **Background information (before coming to Australia)**

1. What was your English learning experiences before coming to Australia and how do you evaluate your English proficiency prior to migration?
2. What was your child’s English learning and Chinese learning before coming to Australia and how do you evaluate your child’s English and Chinese proficiency prior to migration?
3. How about your child’s education in China generally?
4. What kind of job did you do before moving to Australia? How about your life back at home?
5. What made you come to Australia? What was your expectations of your child’s education abroad before coming to Australia?

## Phase 2 Education and adaptation in Australia

## *About parents*

1. How about your experiences of English learning in Australia?
2. What kind of job do you do now?
3. What are your social activities and how about your social circles?
4. Did you see some changes in your life after moving to Australia in terms of your job opportunities, living standard, education opportunities and social activities?
5. What are your happiest experiences (e.g. in education and life) after moving to Australia?
6. What are the problems and difficulties you have experienced in Australia?
7. How much are Chinese values a part of your life (e.g. Chinese language, Chinese books and programs, Chinese food and festivals)?
8. How much are mainstream Australian values a part of your life (e.g. English speaking, English reading and programs, Australian sports, Australian food and festivals)?
9. What do you think of your identity (more Chinese or Australian)?
10. Do you have education goals, career goals and/or other goals for yourself? How do you work towards them?

*About child’s school education*

1. Can you tell me about your child’s school? Why do you choose the school for your child?
2. Did your child receive any language support from school (e.g. ESL program)? What kind of support did he/she receive and what do you feel about it?
3. How about your child’s subject learning in Australian schools? How about your child’s school performance compared with that back home?
4. Does your child go to a tutoring class/coaching college, or have a private tutor or any other support outside school? What’s your reason for sending your child to these classes?
5. How satisfied are you with your child’s education compared with that back home? / What educational aspects are you satisfied and dissatisfied with?
6. Do you see some changes of your expectation towards your child’s education? If so, what kind of changes do you experience and why? What kind of education generally do you want for your child and how do you work towards that?

*About child’s heritage language practice and bilingual abilities*

1. How about your child’s heritage language proficiency and English language proficiency before and after moving to Australia?
2. How do you view the status of your child’s heritage language ability and English language ability?
3. What languages do parents use to the child? What languages does the child use to parents and to siblings? Are there some changes in language use at home and what are those changes? What do your think are the main factors leading to language changes?
4. Do you have family language policy? What’s your family language policy and is it easy or difficult to implement your family language policy?
5. Did you try/Are you trying to maintain your child’s heritage language? What’s the heritage language education goal for your child?
6. Is English language learning an issue in your family? What do your deal with this issue?
7. What is your expectation of your child’s language abilities (bilingual abilities)?

*About child’s social, emotional, and cultural adaptation*

1. In which suburb and/or community are you living? Why do you choose to live there?
2. How about your child’s socializing activities / after-school activities?
3. How important do you view Chinese friends and mainstream Australian friends respectively to your child? What is your child’s social circle?
4. What does your child feel about Australian schools, his friends here and his host country?
5. How important is Chinese culture and mainstream Australian culture respectively to your child? What did you do for that (e.g. festival celebration, book reading, food choices) and what do you think is your child’s cultural identity?
6. What’s the relationship between you and your child? Are there some changes of parent-child relationship between pre- and post-migration? If so, what do you think are the main reasons of these changes?
7. What is your expectation of your child’s identity formation?

**Interview Guides (for child)**

**Phase 1** **Background information (before coming to Australia)**

1. Do you still remember what grade you did finish in China?
2. What subjects did you like when you were in China? How about your school life back in China?
3. How about your English learning in China?
4. What did you usually do outside school? What kind of after-school activities did you enjoy?
5. Can you tell me something about your friends and family members back at home?
6. Who looked after your when you were in China?
7. Did you want to come to Australia? What was your expectations of this new country?

## Phase 2 Life in Australia

## *About social life*

1. Did you like Australia when you first came here and what do you feel now?
2. Can you tell me about your best/worst experience or some funny / embarrassing experiences since coming to Australia?
3. How do you spend your time outside school? What kind of activities do you like more?
4. Do you have some friends here? What languages do you speak to your friends?
5. Do you miss your friends and family members back in China? Do you usually contact them?
6. Who is looking after you in Australia? What languages do you speak to them?

*About languages*

1. How about your English learning in Australia? What kind of language support did you get from and outside your school?
2. Do you still learn your first language? Do you want to improve your first language?
3. What languages does your parents speak to you? What languages do you use to parents and to siblings? What languages do you prefer to use?
4. Do you read English and/or Chinese books? Do you watch English and/or Chinese movies or programs?

*About subject learning*

1. What do you feel about Australian schools?
2. Do you see some differences between your Australian school and your school back in China?
3. What subjects do you like in Australian schools? Are there some subjects which you feel difficult?
4. Do you need to go to a tutoring class or a coaching college? What do you learn there?
5. How much homework do you need to do?

*About future plan*

1. What do you want to do in the future? / What kind of school do you want to go? How do you work towards your dream?
